# *Lactobacillus acidophilus* DDS-1 Modulates Intestinal-Specific Microbiota, Short-Chain Fatty Acid and Immunological Profiles in Aging Mice

**DOI:** 10.3390/nu11061297

**Published:** 2019-06-07

**Authors:** Ravichandra Vemuri, Rohit Gundamaraju, Tanvi Shinde, Agampodi Promoda Perera, Waheedha Basheer, Benjamin Southam, Shakuntla V. Gondalia, Avinash V. Karpe, David J. Beale, Stephen Tristram, Kiran D. K. Ahuja, Madeleine Ball, Christopher J. Martoni, Rajaraman Eri

**Affiliations:** 1 School of Health Sciences, College of Health and Medicine, University of Tasmania, Launceston, Tasmania, 7250 Australia; rohit.gundamaraju@utas.edu.au (R.G.); tanvi.shinde@utas.edu.au (T.S.); agampodi.perera@utas.edu.au (A.P.P.); waheedha.basheer@utas.edu.au (W.B.); benjamin.southam@utas.edu.au (B.S.); stephen.tristram@utas.edu.au (S.T.); kiran.ahuja@utas.edu.au (K.D.K.A.); 2 Centre for Food Safety and Innovation, Tasmanian Institute of Agriculture, University of Tasmania, Launceston, Tasmania, 7250 Australia; 3 Centre for Human Psychopharmacology, Swinburne University of Technology, Hawthorn, Victoria, 3122, Australia; sgondalia@swin.edu.au; 4 Land and Water, Commonwealth Scientific and Industrial Research Organization (CSIRO), Ecosciences Precinct, Dutton Park, Queensland, 4102, Australia; avinash.karpe@csiro.au (A.V.K.); david.beale@csiro.au (D.J.B.); 5 School of Health and Biomedical Sciences, RMIT University, Bundoora, Victoria, 3082 Australia; madeleine.ball@utas.edu.au; 6 UAS Laboratories, Madison, Wisconsin, 54401 WI, USA; cmartoni@uaslabs.com

**Keywords:** intestinal microbiota, aging, probiotics, short-chain fatty acids, immune responses

## Abstract

Distribution of the microbiota varies according to the location in the gastrointestinal (GI) tract. Thus, dysbiosis during aging may not be limited to faecal microbiota and extend to the other parts of the GI tract, especially the cecum and colon. *Lactobacillus acidophilus* DDS-1, a probiotic strain, has been shown to modulate faecal microbiota and its associated metabolic phenotype in aging mice. In the present study, we investigated the effect of *L. acidophilus* DDS-1 supplementation on caecal- and mucosal-associated microbiota, short-chain fatty acids (SCFAs) and immunological profiles in young and aging C57BL/6J mice. Besides differences in the young and aging control groups, we observed microbial shifts in caecal and mucosal samples, leading to an alteration in SCFA levels and immune response. DDS-1 treatment increased the abundances of beneficial bacteria such as *Akkermansia* spp. and *Lactobacillus* spp. more effectively in caecal samples than in mucosal samples. DDS-1 also enhanced the levels of butyrate, while downregulating the production of inflammatory cytokines (IL-6, IL-1β, IL-1α, MCP-1, MIP-1α, MIP-1β, IL-12 and IFN-γ) in serum and colonic explants. Our findings suggest distinct patterns of intestinal microbiota, improvements in SCFA and immunological profiles with DDS-1 supplementation in aging mice.

## 1. Introduction

The global proportion of aging individuals above 60 years old is estimated to increase from 11–12% currently, to more than 25% by 2050 [1,2]. Although life expectancy has generally increased with advances in public health and medicine, there is little evidence of any concurrent increase in the overall health of aging individuals [3,4]. Aging is characterized by a decline in physiological functions of the human body [1,5]. Altered gastrointestinal (GI) physiology affects the nutritional status of aging individuals by impacting the host’s metabolism as well as their gut microbiota [5,6]. Therefore, it is essential to comprehend dietary and nutrition-related changes in aging individuals to provide dietary mechanisms to promote healthy aging.

The gut microbiota plays a fundamental role in maintaining the overall health of individuals via interacting with the immune system and host metabolism. Gut microbiota is established during birth and evolves with age, mostly remaining stable through adulthood [5,7,8]. However, a few human and animal studies suggest a major shift in the gut microbial composition in aging populations when compared to younger groups [6,9,10]. Recent studies have reported that Bacteroidetes, Firmicute, and Verrucomicrobia are overrepresented in younger individuals, but their abundance tends to decrease with age [5,11,12,13]. Moreover, during such microbial shifts, bacteria belonging to the Enterobacteriaceae family (Proteobacteria) increased, which has been implicated in the aetiology of various GI and metabolic diseases [9,14,15].

Most of the previous studies have investigated the microbial profile in faecal samples. However, very recently, differences of microbiota along the GI tract have been reported in human and animal studies, suggesting a likely distinction between faecal and mucosal-associated microbiota [16,17,18]. This could be due to an increase of bacterial load from the stomach to the colon [7,16]. Along these lines, a few studies have indicated differences in the caecal and faecal microbiota, which lead to alteration in metabolic profiles in mice [19,20,21]. In a study on aging rats, the abundance of Lactobacillaceae in the ileum and Ruminococcaceae and Lachnospiraceae in the cecum and faeces were higher compared to the control young group [16]. From the studies mentioned above, it can be speculated that dysbiotic shifts may extend to the other parts of the GI tract, especially the cecum and colonic-mucosa, where the microbial concentration is at a higher level [7].

Concomitantly with microbiota shifts, immunity becomes impaired in aging individuals [22]. The decline in immune responses or low-grade chronic inflammation, known as “inflamm-aging” is a hallmark of aging [23]. During this condition, homeostasis between pro-inflammatory cytokines and the regulatory response (microbes-immune system axis) is disoriented, which is likely to contribute to the pathophysiology of various GI diseases [22,23,24]. Gut microbiota also produces short-chain fatty acids (SCFAs) which regulate intestinal barrier integrity and immune homeostasis [25]. In human and animal studies, the SCFA levels were found to be decreased with an imbalance in gut microbiota, affecting immune homeostasis [3,26,27]. Hence, for healthy aging the maintenance of proper microbial populations of caecal and mucosal sites is essential.

Probiotics are living microbes that, when administered in adequate amounts, confer health benefits on the host [5,14]. In a variety of animal models, treatment with probiotics containing lactic acid bacteria (LAB) have been reported to modulate the gut microbiota, SCFA production and inflammatory responses [6,21,27]. Previously, we have reported that *L. acidophilus* DDS-1, a clinically-documented probiotic strain [28,29,30,31], was able to improve the metabolic phenotype via modulating faecal microbiota composition in aging mice [6]. Therefore, we hypothesized that this probiotic *L. acidophilus* DDS-1 could also enhance the beneficial microbial composition of caecal and mucosal-associated microbiota in aging.

In the present study, we employed a healthy-aging-based mice model to investigate the dynamic effect of caecal- and mucosal-associated microbiota in aging. A combination of 16S rRNA gene sequencing, untargeted metabolomics of volatile fatty acids and cytokine measurement with Bio-plex-based immunological analysis were used to provide a comprehensive understanding of the potential beneficial effects of DDS-1 on the intestinal microbial changes in aging mice.

## 2. Methods and Material

### 2.1. Ethics Statement

All animal experiments and procedures were approved by the Animal Ethics Committee of the University of Tasmania (Tasmania, Australia), and the entire study was performed in compliance with 8th edition (2013) of the Australian Code of Practice for the Care and Use of Animals for Scientific Purposes of the National Health and Medical Research Council (ethics identification number: A0015840).

### 2.2. Bacterial Culture and Probiotic DDS-1 Viability in the Feed

The bacterial strain utilized in the study, *L. acidophilus* DDS-1, was obtained in freeze-dried, free-flowing lyophilized form from UAS labs, Madison, WI, USA as described previously [6]. The bacterial culture was suspended in saline before making the probiotic chow (by mixing both) at a concentration of 3 × 10^9^ colony forming units (CFU)/g. The viability of the probiotic DDS-1 in the chow was assessed at the following intervals over a 24 h period (at 0 min, 1 h, 2 h, 5 h, and 24 h) and formulated to deliver 3 × 10^9^ CFU/g as shown by Vemuri et al. and Kuo et al. [6,32].

### 2.3. Animals and Study Design

Thirty-two C5BL/6J mice including young mice 3–4 weeks old (*n* = 16) and aging mice (*n* = 16) 35–36 weeks old with average weights of 19 and 25 g, respectively were obtained from the Univerity of Tasmania (UTAS) animal breeding facility. All mice were kept in a temperature-contained environment with a 12 h day–night light cycle and individually caged throughout the study. Radiation-sterilized rodent feed pellets (Barastoc Rat and Mouse, Ridley Agriproducts, Melbourne, Australia) and distilled water were available to all mice ad libitum. After a week of acclimatization, all mice were divided into the following four groups based on their age and treatments: 1) young control (YC), 2) young probiotic, 3) aging control, and 4) aging probiotic. Mice in YC and AC groups were fed with normal chow pellets (4 g). Each mouse in the YP and AP groups received (4 g) chow mash supplemented with *L. acidophilus* DDS-1 probiotic at 3 × 10^9^ CFU/g/day. As mentioned in the methods Section 2.2, the chow mash (with and without probiotics) was prepared fresh each day throughout the study.

### 2.4. Clinical Parameters and Sample Collection

The body weight of each mouse was recorded throughout the study. All the animals were sacrificed utilizing CO_2_ asphyxiation at the end of the study and all efforts were taken to minimize the suffering of the animals. Subsequently, the colons of mice were removed from the cecum to the anal end as previously described [33]. The caecal content was collected by following the methods of Sybille et al. [34]. Briefly, the cecum was removed from the colon and the cecum was dissected in the longitudinal axis and caecal content was collected by sterilized pipette tips using the scraping method. To collect the luminal or mucosal content, the colon was dissected in a longitudinal axis and the luminal content was collected utilizing a sterilized pipette tip by following the scrapping method of Lamoureux et al. [35]. The luminal contents of cecum and colonic-mucosa were carefully collected in at least two sets from each mouse and immediately transferred into a sterile microcentrifuge tube. These samples were immediately stored at −80 °C for subsequent 16S rRNA gene sequencing and metabolomic analysis. Colon tissues were excised and snap frozen immediately before further analysis.

### 2.5. Histological Analysis

The length of each colon was recorded before its longitudinal bisection. The swiss roll method was used, and sections of the colon were stained with haematoxylin and eosin (H&E; HD Scientific, Sydney, Australia) as described previously [36]. Alcian blue (Ab) staining was used to identify acidic carbohydrates and periodic acid schiff (PAS) for neutral carbohydrates, both of which occur on the MUC2 glycoprotein. Alcian blue staining and the measure of their staining intensities were assessed by the method described previously [37].

### 2.6. Serum Collection

Blood from each individual mouse was collected by cardiac puncture at the end of the treatment into vacutainer tubes containing no anticoagulant. The vacutainer was incubated in an upright position at room temperature for 30–45 min to allow clotting, then centrifuged at 3000× *g* for 15 min, and the supernatant (serum) was collected in cryovials and stored at −80 °C.

### 2.7. Colonic Tissue Explant Culture

The proximal and distal ends of each colon were cut and washed with phosphate-buffered saline (PBS) before placing them in RPMI 1640 culture medium as described previously [33].

### 2.8. Cytokine Measurement

For measurement of the cytokine levels in colonic tissues and serum a Bio-Plex Pro Mouse cytokine 23-plex kit (Bio-Rad Laboratories, Inc., Hercules, CA, USA) was utilized following the manufacturer’s instructions. A Bio-Plex 200 instrument and a Bio-Plex Manager software, version 6 were used to analyse the obtained cytokine concentrations. The cytokine levels were normalized by following the method of Perera et al. [33]. The results of serum cytokine levels were expressed in pg/ml and colonic tissue cytokine levels were expressed as pg/mL/g of tissue.

### 2.9. Microbiota Analysis Using 16s rRNA High-Throughput Sequencing

DNA was isolated the from caecal (*n* = 5) and mucosal (*n* = 5) samples using the QIAamp DNA Stool Mini Kit (Qiagen, Melbourne, VIC, Australia). A high-throughput sequencing on the Illumina MiSeq platform for each sample was performed at the Australian Genome Research Facility (University of Queensland, Brisbane, QLD, Australia). Hypervariable regions of V3–V4 of bacterial 16S rRNA genes were sequenced and data obtained, assigned and analysed as described previously [6]. 16S rRNA gene sequences were analysed using MEGAN6 (Community edition version) [38], Microbiome analyst [39] and QIIME. Statistical analysis of Bray–Curtis dissimilarities was calculated using the relative abundances of bacterial genera using Adonis function in R (version 3.2).

### 2.10. Metabolomics Analysis

All the samples (caecal and mucosal) were prepared and derivatized following the protocol developed by Furuhashi et al. [40] with some modifications. Briefly, caecal (*n* = 5) and mucosal (*n* = 5) samples (stored at −80 °C) were weighed to ± 0.1 mg accuracy. These samples (100–150 mg fresh weight) were added to a sterile 1.5 mL bead-beating tube (NAVY Rino Lysis tubes, Next Advance, Troy, NY, USA). Isobutanol (10% in water, volume = 1.0 mL, LC-MS grade, Merck, Castle Hill, NSW, Australia) was added to each sample, followed by two 30 s, 4000 rpm homogenization pulses sandwiched between a 20 s pause interval (Precellys Evolution Homogenizer, Bertin Instruments, Montigny-le-Bretonneux, France). The samples were subsequently centrifuged at 15,700 g for 6 min.

The supernatant (675 µL) was transferred to a clean round-bottomed 2 mL centrifuge tube (Eppendorf South Pacific Pty. Ltd., Macquarie Park, NSW, Australia), and NaOH (20 mM, 125 µL, Merck, Castle Hill, NSW, Australia) and chloroform (400 µL, LC-MS grade, Merck, Castle Hill, NSW, Australia) were added. The samples were briefly vortexed and centrifuged at 15,700 rpm for 3 min. The aqueous phase (upper layer, 400 µL) was transferred to a new clean round-bottomed 2 mL centrifuge tube (Eppendorf South Pacific Pty. Ltd., Macquarie Park, NSW, Australia) containing a boiling chip (Sigma Aldrich, Castle Hill, NSW, Australia). Pyridine (100 µL), isobutanol (80 µL) (both LC-MS grade, Sigma Aldrich, Castle Hill, NSW, Australia) and milliQ water (70 µL) were added and the samples were subjected to gentle hand vortexing (swirling action) followed by the addition of 50 µL isobutyl chloroformate (assay = 98%, Sigma Aldrich, Castle Hill, NSW, Australia). The tube was kept opened to release any generated gases and was allowed to stand for about 1 min. Hexane (150 µL, LC-MS grade, Sigma Aldrich, Castle Hill, NSW, Australia) was then added to each tube, which was capped and vortexed prior to centrifugation at 15,700 g for 4 min. The upper phase (100 µL) was subsequently transferred to clean GC autosampler vials fitted with salinized glass inserts; malathion (1 µL, equivalent to 2.5 µg/mL dry weight) was added as an internal standard.

The GC-MS analysis was performed on an Agilent 6890B gas chromatograph (GC) oven coupled to a 5977B mass spectrometer (MS) detector (Agilent Technologies, Mulgrave, VIC, Australia) fitted with an multi purpose (MPS) autosampler (Gerstel GmbH and Co.KG, Mülheim an der Ruhr, Germany). The GC oven was fitted with two 15 m HP-5MS columns (0.25 mm ID and 0.25 µm film thickness; 19091S-431 UI, Agilent Technologies, Mulgrave, VIC, Australia), coupled to each other through a purged ultimate union (PUU) for the use of post-run back-flushing. The sample (1.0 µL) was introduced via a multimode inlet (MMI) operated in split mode (1:20). The column was maintained at 40 °C for 5 min, followed by an increase to 250 °C at a rate of 10 °C/min. This was followed by a second increment to 310 °C at a rate of 60 °C/min. The column was held at 310 °C for 1 min. The mass spectrometer was kept in extractor ion mode (EI mode) at 70 eV. The GC-MS ion source temperature and transfer line were kept at 250 and 280 °C, respectively. Detector voltage was kept at 1054 V. The MS detector was turned off for the first 3 min and, at 4.0–4.8 min and 12.5–13.2-min time windows until the excess derivatization reagent and chloroformate/hexane solvents were eluted from the column. This ensured that the source filament was not saturated and damaged. The scan range was kept in the range of *m/z* 35–350 (35–350 Daltons). Data acquisition and spectral analysis were performed as described in our previous study [6] and qualitative identification of metabolites was performed according to the Metabolomics Standard Initiative (MSI) chemical analysis workgroup [41] using standard GC-MS reference metabolite libraries (NIST 17, Agilent Fiehn RTL Library [G166766A, Agilent Technologies] with the use of Kovats retention indices based on a reference n-alkane standard (C8-C40 Alkanes Calibration Standard, Sigma-Aldrich, Castle Hill, NSW, Australia).

### 2.11. Multivariate and Statistical Analysis

To determine the overall microbial variation in the four groups, a non-metric multidimensional scaling (NMDS) with permutational multivariate analysis of variance statistical method and a principal coordinate analysis (PCoA) were used with the Bray–Curtis ecological indexing and Euclidean distances as the similarity measure, and Ward’s linkage as a clustering algorithm as described previously [6]. Data are presented as mean values ± standard error from multiple individual experiments, each carried out in triplicate measurements in a representative experiment. Graph Pad Prism version 7.0 for Windows was used for the statistical analysis. Statistical analyses were done using an unpaired two-tailed t-test for comparison between the two groups in the study. The data were evaluated with one-way analysis of variance (ANOVA) and using Tukey’s test for multiple comparisons with a statistical significance of *p* < 0.05. For comparative microbial analysis, a linear discriminant effect size (LEfSe) analysis was performed (α = 0.05), logarithmic linear discriminant analysis (LDA) score threshold = 1.0.

## 3. Results

### 3.1. DDS-1 Viable in the Diet after 24 h and Exerts an Effect on Colon Lengths and Colon Weights of Young DDS-1 Treated Mice Only but Not on Body Weights and Spleen Weights 

**Figure 1:**
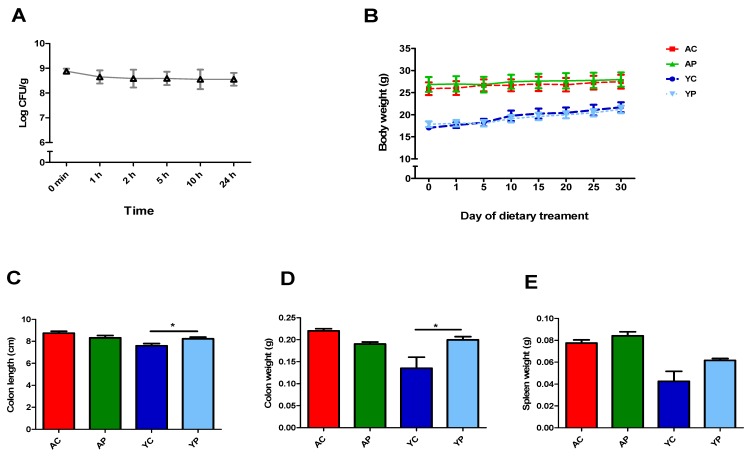
*L. acidophilus* DDS-1 viability in the diet during 24 h of storage (**A**). The effect of DDS-1 supplementation on body weights (**B**), colon length (**C**), colon weight (**D**) and spleen weights (**E**) observed in young control (YC), young probiotic group (YP), aging control group (AC) and aging probiotic group (AP) * *p* < 0.001 (one-way ANOVA with Tukey’s post-hoc test and data are expressed as the mean ± SEM).

The viability of DDS-1 in the chow was maintained, and no significant loss was observed even at the 24 h time point (Figure 1A). There was no significant difference in body weights and spleen weights (Figure 1B,E). The colon weights of the YP group (*p* < 0.001) were significantly different when compared to the YC group (Figure 1D). The colon length was different only in the YP group (*p* = 0.02) when compared to the YC group (Figure 1C).

### 3.2. DDS-1 Supplementation Improves Goblet Cell Structure

**Figure 2:**
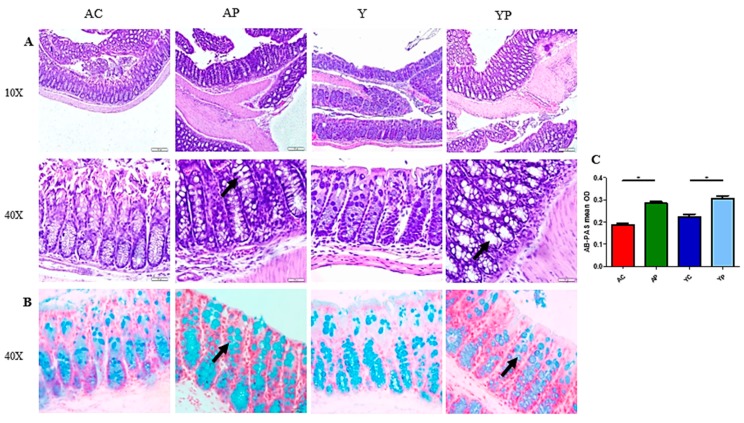
Representation of distal colon sections stained with haematoxylin and eosin (**A**) and AB-PAS staining (**B**) for sulphated mucins among the four groups. (**C**) The AB-PAS staining mean optical density of goblet cells observed in the young control group (YC), young probiotic group (YP), aging control group (AC) and aging probiotic group (AP). Magnification is reported, and the arrow indicates location goblet cells. * *p* < 0.005 (one-way ANOVA with Tukey’s post-hoc test and data are expressed as the mean ± SEM).

The colonic H&E staining showed normal anatomical architecture. However, there was a difference in the size and number of goblet cells (Figure 2A) in the DDS-1 treated groups. Noticeable changes in Ab-PAS-positive goblet cells were observed in the colon of mice in both YP and AP groups (Figure 2B). In addition, the mean optical density (OD) of both YP and AP groups (OD = 0.32 ± 0.04) were significantly higher than that of the control groups (OD = 0.20 ± 0.03, *p* < 0.05) (Figure 2C). The mucus layer was significantly thicker in YP and AP groups compared with the control groups.

### 3.3. DDS-1 Supplementation Reduces Local and Systemic Pro-Inflammatory Cytokine Levels

**Figure 3:**
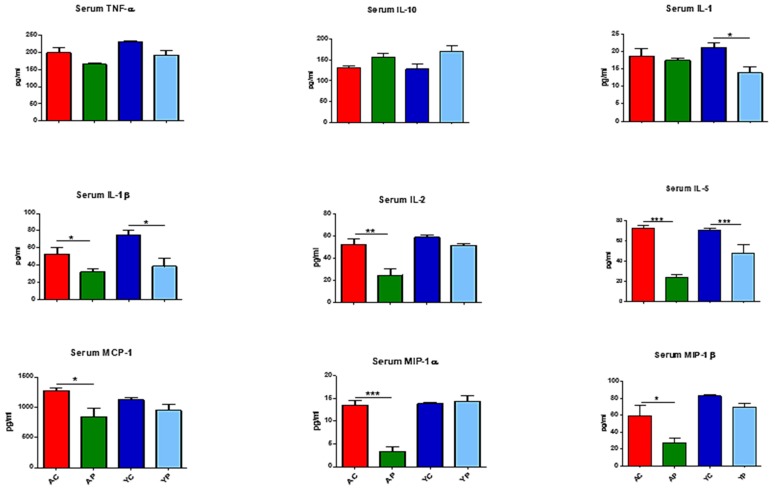
Effect of *L. acidophilus* DDS-1 supplementation on serum cytokine levels observed in the young control group (YC), young probiotic group (YP), aging control group (AC) and aging probiotic group (AP). * *p*  <  0.05, ** *p*  <  0.01, *** *p*  <  0.001 (one-way ANOVA with Tukey’s post-hoc test and data are expressed as the mean ± SEM).

The serum cytokine analysis detected certain pro-inflammatory cytokines such as TNF-α, IL-1β, IL-1α, IL-2, IL-5, MCP-1, MIP-1α, MIP-1β and an anti-inflammatory cytokine, IL-10, in the four groups (Figure 3). Of these, IL-6, IL-1β, IL-1α, MCP-1, MIP-1α and MIP-1β showed differences with DDS-1 treatment. Relative to the YC group, the levels of IL-1α (from 21.2 ± 2.2 pg/mL to 13.8 ± 4.30 pg/mL, *p* = 0.053) and IL-1β (74.38 ± 9.38 pg/mL to 38.73 ± 19.37 pg/mL, *p* = 0.01) were significantly lower in the YP group. Similarly, IL-2 (from 52.49 ± 8.51 to 24.74 ± 9.14 pg/mL, *p* = 0.01), MCP-1 (from 1271 ± 100 to 844.34 ± 212.66 pg/mL, *p* = 0.004), MIP-1α (from 13.46 ± 2.12 to 3.33 ± 1.39 pg/mL, *p* = 0.0001) and MIP-1β (59.74 ± 22.56 to 27.01 ± 9.17 pg/mL, *p* = 0.002) levels were significantly downregulated in the AP group compared to the AC group. Furthermore, IL-5 levels were downregulated in both the AP (70.03 ± 4.41 pg/mL, *p* = 0.003) and YP (39.28 ± 23.75 pg/mL, *p* = 0.003) groups compared to the AC and YC groups, respectively. Although not significant, TNF-α levels (from YC to YP: 229.07 ± 6.51 to 191.89 ± 23.4 pg/mL; AC to AP: 197.94 ± 31.6 to 164.19 ± 8.04 pg/mL) were notably reduced and IL-10 levels (from YC to YP 127.59 ± 23.27 to 171.59 ± 18.05 pg/mL; AC to AP: 131.26 ± 7.83 to 157.19 ± 8.41 pg/mL) were altered following treatment with DDS-1 in the YP and AP groups, compared to the YC and AC groups, respectively.

**Figure 4:**
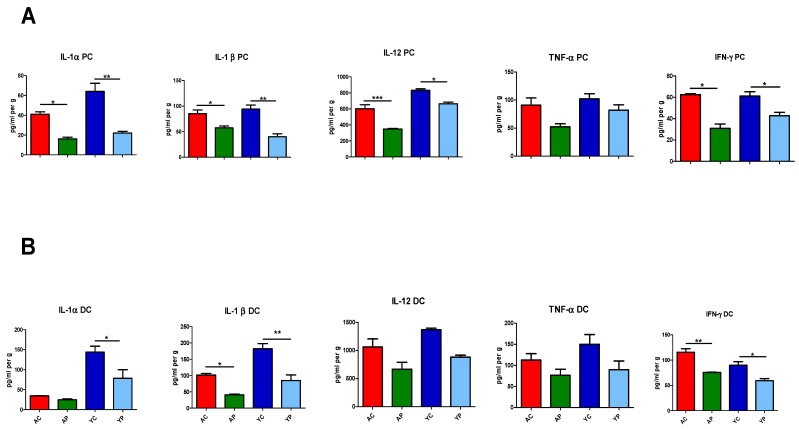
Effect of *L. acidophilus* DDS-1 supplementation on colon explants of the proximal-(PC) (**A**) and distal colon (DC) (**B**) observed in the young control group (YC), young probiotic group (YP), aging control group (AC) and aging probiotic group (AP). * *p* <  0.05, ** *p* < 0.01, *** *p* <  0.001 (one-way ANOVA with Tukey’s post-hoc test comparisons and data expressed as the mean ± SEM).

At the tissue level, certain pro-inflammatory cytokines such as IL-1α, IL-1β, IL-12 and IFN-γ were detected in proximal and distal ends of the colon in the four groups (Figure 4A,B). At the proximal end, DDS-1 treatment in the YP group modulated the levels of IL-1α from 64.01 ± 14.87 to 22.09 ± 3.27 (*p* = 0.0003), IL-1β from 94.31 ± 13.78 to 40.03 ± 10.71 (*p* = 0.0005), IL-12 from 831.44 ± 22.66 to 664.47 ± 32.88 (*p* = 0.006) and IFN-γ from 61.15 ± 7.85 to 42.72 ± 6.18 (*p* = 0.01), when compared to the YC group. Similar changes were observed in the distal end of the YP group relative to the YC group. The basal levels of IL-1α, IL-1β, IL-12 and IFN-γ were (pg/mL per g) 143.86 ± 23.34, 143.86 ± 23.34, 1368.1 ± 27.4 and 89.92 ± 6.93, and DDS-1 downregulated these levels in the YP group to 78.47 ± 24.03 (*p* = 0.01 ), 84.89 ± 33.53 (*p* = 0.001), 880.3 ± 34.7 (*p* = 0.04) and 59.33 ± 4.67 (*p* = 0.01), respectively. Among aging mice, at the proximal end, DDS-1 was shown to downregulate the levels of IL-1α from 41.07 ± 4.59 to 16.26 ± 1.90 (*p* = 0.008), IL-1β from 85.48 ± 10.14 to 57.56 ± 6.93 (*p* = 0.025), IL-12 levels from 602.04 ± 105.46 to 347.43 ± 11.73 (*p* = 0.0001) and IFN-γ levels from 62.47 ± 1.03 to 30.92 ± 7.68 (*p* = 0.0003) (from the AC to AP groups). However, at the distal end in the AP group, DDS-1 only modulated IL-1β (101.29 ± 7.67 to 40.93 ± 3.10, *p* = 0.01) and IFN-γ (112.33 ± 13.67 to 75.66 ± 1.34, *p* = 0.001) compared to the AC group.

### 3.4. Distinct Modulation of Intestinal Microbiota with DDS-1 Supplementation

#### 3.4.1. Changes at the Phylum Level

**Figure 5:**
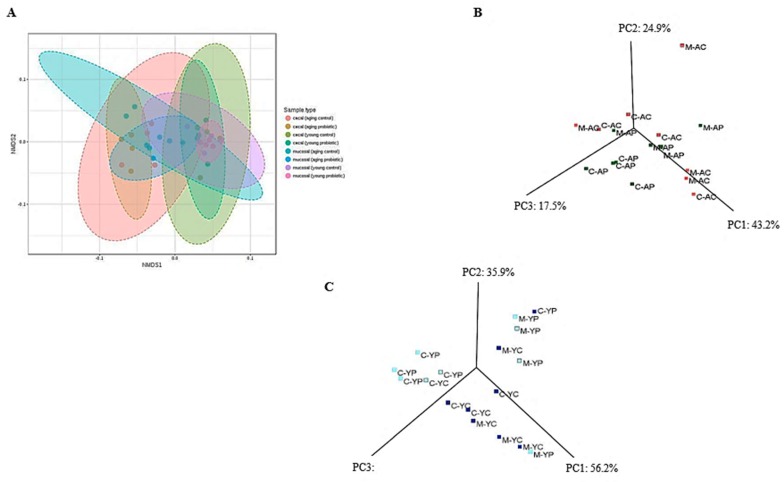
Caecal- and mucosal-associated microbiota changes observed in the young control group (YC), young probiotic group (YP), aging control group (AC) and aging probiotic group (AP) differentiated by (**A**) non-metric multidimensional scaling (NMDS). Principal coordinate (PC) analysis (PCoA) plot showing the differentiation between aging (**B**) and young (**C**) mouse groups for both caecal (**C**) and mucosal (M) samples.

**Figure 6:**
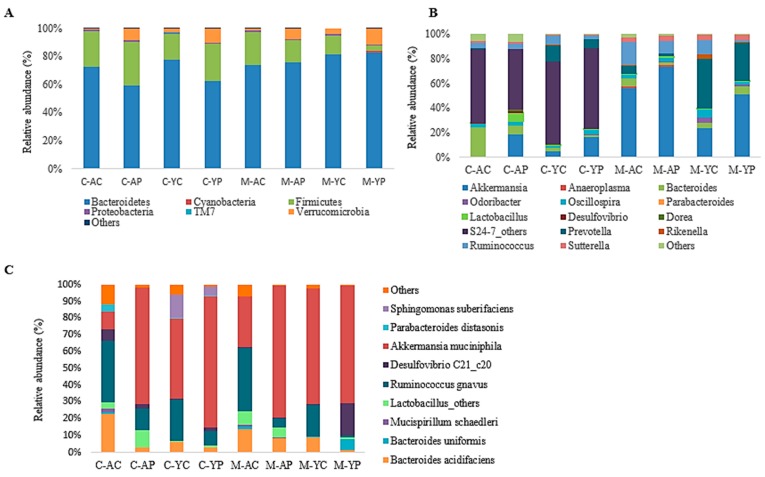
Relative abundances (%) of caecal and mucosal-associated microbiota at phylum (**A**)**,** genus (**B**) and species levels (**C**) observed in the young control group (YC), young probiotic group (YP), aging control group (AC) and aging probiotic group (AP). Caecal (C); mucosal (M).

The taxonomic and functional profiles of 32 samples (*n* = 6 per group) including the caecal and mucosal (luminal) content of all controls and DDS-1 supplemented groups were generated using the 16S rRNA gene sequencing-based method. To compare the changes among the groups, NMDS and PCoA were used. NMDS (F-value = 2.438, R^2^ = 0.415, *p* < 0.001, NMDS stress = 0.149) and PCoA plots of phylogeny showed a clear separation of each group with three distinct clusters at the operational taxonomic units (OTU) level among the four groups (Figure 5A–C) suggesting that DDS-1 modulated caecal and mucosal microbiota. Notably, the inter-individual variation was higher among mucosal samples compared to caecal samples in all four groups. That is, while microbial communities of mucosal samples were scattered, those of caecal samples were more closely gathered. Figure 6A indicates the phylum-level changes in the caecal and mucosal microbiota, which is significantly dominated by Bacteroidetes and Firmicutes and moderately dominated by Verrucomicrobia.

#### 3.4.2. Caecal Microbiota

**Figure 7:**
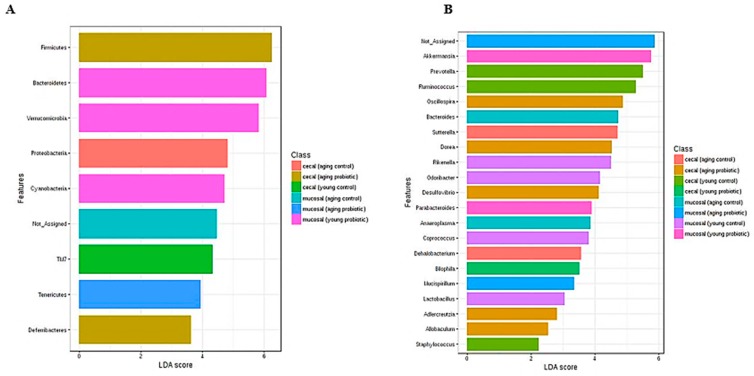
Biomarker analysis with linear discriminant analysis (LDA) effect size (LEfSe) scoring plot using the Kruskal–Wallis rank sum test. Aging and young mouse groups at phylum (**A**) and genus (**B**) levels.

Around 99% of the total microbial abundance was classified into six major phyla, while the rest were allocated as unclassified or others, as shown in the LEfSe scoring plot (Figure 7A). DDS-1 increased the abundance of Firmicutes and decreased the abundance of Bacteroidetes. In caecal samples in young mice, Firmicutes levels were increased from 19.58% to 28.30% and Bacteroidetes levels were reduced from 75.40% to 60.80% (from the YC to YP group) (Figure 6A). Similarly, in aging mice, when comparing the AC and AP groups, the Firmicutes abundance (25.50% to 32.8%) was increased, while Bacteroidetes abundance (69.30% to 63.60%) was reduced.

#### 3.4.3. Mucosal Microbiota

Regarding the mucosal samples, the DDS-1 treatment in the YP group marginally increased the abundance of Bacteroidetes from 81.10% to 83% and decreased Firmicutes levels from 13.50% to 3.80% when compared to the YC group (Figure 6A). The abundance of Bacteroidetes in the AP group was increased from 68.20% to 80.70%, while Firmicutes levels were decreased from 22.68% to 17.53% when compared to the AC group. Additionally, Verrucomicrobia abundance in caecal and mucosal microbiota was significantly increased in both treatment groups when compared to respective control groups (*p* < 0.05).

#### 3.4.4. Specific Beneficial Alterations at the Genus and Species Level

At the genus level, the distribution of microbial populations of aging mice was markedly different when compared to young mice, in both caecal and mucosal microbiota (Figure 6B). In caecal and mucosal microbiota, the DDS-1 treatment enriched the populations of *Akkermansia*, *Lactobacillus, Odoribacter*, *Oscillospira* and *Rikenella* while reducing the abundances of *Prevotella*, *Oscillospira* and *Ruminococcus* in the YP and AP compared to the YC and AC groups, as shown by LEfSe analysis (Figure 7B). *Parabacteroides* and *Anaeroplasma* were undetected in the caecal microbiota, and *Desulfovibrio* and *Dorea* were undetected in mucosal microbiota in all four groups. Interestingly, the relatively new family S24-7 of the phylum *Bacteroidetes* were only present in caecal microbiota. At the species level, DDS-1 treatment significantly increased *A. muciniphila* (*p* < 0.05) levels, and decreased *Ruminococcus gnavus*, *Parabacteroides distasonis*, *Bacteroides acidifaciences* and *Bacteroides uniformis* levels but the differences were not statistically significant (Figure 6C).

#### 3.4.5. A Significant Increase in the Production of SCFAs

**Figure 8:**
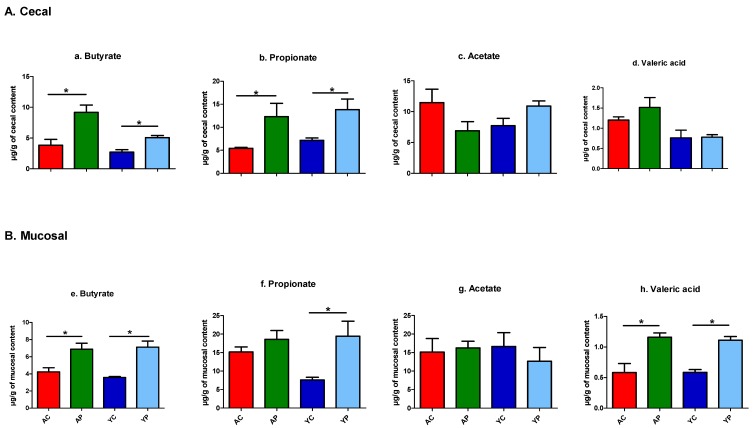
Short-chain fatty acid (SCFA) distribution observed in the young control group (YC), young probiotic group (YP), aging control group (AC) and aging probiotic group (AP). The concentrations of butyrate, propionate, acetate and valeric acid in the caecal (*n* = 5) (**A**) and mucosal (*n* = 5) (**B**) samples were measured by GC-MS analysis from caecal and mucosal contents at the end of the study. * *p*  <  0.05 (One-way ANOVA with Tukey’s post-hoc test and data are expressed as the mean ± SEM).

A total of 25 volatile compounds were identified in our untargeted metabolomics from 46 cecum and mucosal samples in all four groups (Tables S1 and S2). These 25 metabolites included the three major SCFAs of butyrate, propionate and acetate. DDS-1 differentially elevated butyrate, propionate, and acetate in the cecum and mucosal samples across groups (Figure 8A,B). In both caecal and mucosal samples, the butyrate and propionate levels were significantly increased in both the YP and AP groups compared to the YC and AC groups, respectively. There was no change observed in acetate levels in caecal and mucosal samples with DDS-1 treatment. Notably, valeric acid levels were upregulated in mucosal samples with DDS-1 treatment in both the YP and AP groups.

## 4. Discussion

Distribution of the microbiota varies according to the location in the GI tract [1,3,11,16,17]. Thus, dysbiosis during aging may not be limited to faecal microbiota and may extend to the entire GI tract, leading to alterations in metabolic profile and immune responses [6,24,42]. Therefore, the present study aimed to evaluate the comparison of caecal and mucosal-associated microbiota, SCFA production and immune responses with *L. acidophilus* DDS-1 supplementation in relation to age (young and aging), utilizing 16S rRNA gene sequencing analysis and untargeted metabolomics of volatile fatty acids. Besides differences in the young and aging control groups, our study confirms microbial shifts in caecal and mucosal samples. The taxa showing altered abundance might mediate dysbiosis in aging mice. Importantly, treatment with DDS-1 increased the abundance of specific bacterial species such as *A. muciniphila,* helped modulate the overall intestinal microbiota, increased butyrate production and reduced pro-inflammatory responses.

In our study, high-throughput analysis of the caecal and luminal (mucosal) microbiota revealed noticeable changes in microbial communities. Bacteroidetes, Firmicutes and Verrucomicrobia were the most dominant phyla across age groups, which was consistent with our previous study [6]. We observed greater inter-individual variation in mucosal samples when compared to caecal samples. Such differences can be explained by the transient nature of mucosal-associated microbiota, as described by Lee et al. [16], and are consistent with previous similar studies on young/old mice and rats [13,17,21,42,43]. Interestingly, DDS-1 had differential effects on caecal and mucosal microbiota at the phylum level. Similar to faecal microbiota profiles [6], DDS-1 increased Firmicutes in both age groups in caecal samples, reflecting the role of *Lactobacillus* spp. in overall abundance.

In contrast, Bacteroidetes showed modest increases post-DDS-1 among mucosal samples. We speculate this change could be due to the transient nature of mucosal-associated intestinal microbiota and needs further examination. Although Verrucomicrobia was present in all four groups of caecal and mucosal samples, their relative abundance was higher in the DDS-1 treated groups. It is noteworthy that more than 50% of mouse cecum was enriched by the relatively new family S24-7, which belongs to the Bacteroidetes phylum, and is consistent with our previous study [6]. However, S24-7 was completely undetected in mucosal samples. The consequences of these fluctuations in abundance of S24-7 is unknown, and a few researchers have hypothesized that they could play a role in butyrate production [44,45].

At the genus and species level, *Lactobacillus* levels were mostly enriched in aging mice administered DDS-1. Notable changes in this study were an increase of *R. gnavus* and *B. acidifaciens* in aging mice caecal and mucosal samples when compared to younger mice. Such differences were in line with previously reported faecal microbiota analysis [6,46]. The role of the bacteria such as *R. gnavus* and *B. acidifaciens* in inflammatory bowel diseases was highlighted previously. [15,46]. Both these bacteria are mucin degraders and may play an essential role in dysbiosis. DDS-1 was shown to reduce the abundance of both *R. gnavus*, and *B. acidifaciens* and thus could play a role in reducing inflammation. *A. muciniphila* levels were increased by DDS-1, irrespective of age, and could be related to improvement in metabolic profiles. These changes are consistent with previous studies on young/aging mice [6,12,21,46].

The gut microbiota produces SCFAs, which ameliorate host energy metabolism and inflammation [42]. A series of animal studies have previously demonstrated that dietary supplementation with probiotics of *Lactobacillus* spp. have induced SCFA production by modulation of gut microbiota [3,26,27,46]. Specifically, the phylum Bacteroidetes members mainly produce acetate and propionate, whereas the phylum Firmicutes have butyrate as its primary metabolic end product [47]. Consistent with previous studies, butyrate production was increased in caecal samples across age groups in treated mice and was shown to be directly correlated with an increase in Firmicutes levels [3,27]. Butyrate is a primary source of energy for colonocytes, and it has been suggested to play a role in the prevention and treatment of distal ulcerative colitis, Crohn’s disease and cancer [46]. Previously, a study by Lee et al. [11] demonstrated that butyrate improved mucin production, which was also regulated by *A. muciniphila*, and the mucus layer thickness was highly related with metabolic improvement, consistent with mucin production in the present study. Propionate levels were also found to be increased in both DDS-1 treated groups of caecal and mucosal samples, and it could also be positively correlated with increased *Akkermansia* [34]. Distinct from the other SCFAs; however, there is only a little evidence about the role of valeric acid on the gut health. A limited number of studies have reported that valeric acid may stimulate intestinal growth and ameliorate the pathogenesis of diseases ranging from colitis and cancer to cardio-metabolic diseases [48,49,50]. Moreover, a variety of animal and human studies have reported very low to undetectable concentrations of valeric acid in faeces, colon, cecum, and ileum [51,52,53,54]. Interestingly, in the present study, valeric acid levels were clearly detected and upregulated among mucosal samples in both DDS-1 treated groups and were positively correlated with an increase in Bacteroidetes level [52]. Further research is needed to understand the role of valeric acid in gut health parameters.

Age-related alteration in tissue and circulating cytokine production contributes to low-grade chronic inflammation and release of pro-inflammatory factors such as nuclear factor-kappa B (NF-κB) [1,3,22,23,24]. Pro-inflammatory cytokines such as IL-1α, IL-1β, IL-2, IL-5, IL-6, IFN-γ, MCP-1, MIP-1α, MIP-1β and TNF-α are shown to play an essential role in inducing age-related chronic inflammation [22,23,24,55]. Notably, studies on aging mice have suggested an imbalance in cytokine type 1 T helper (h) cell/type 2 T helper cell production to be associated with chronic inflammation [55,56,57]. This could be due to imbalances in the intestinal microbiota that lead to compromised gut-barrier integrity, enhanced inflammatory responses and the subsequent development of chronic diseases [22,58]. For example, the segmented filamentous bacteria have been involved in the induction of Th cell subsets in the gut [1,59]. In contrast, polysaccharide A produced by *Bacteroides fragilis* and certain *Clostridium* spp. have shown to induce anti-inflammatory IL-10 releasing T regulatory cells in the gut [7]. Consistent with these studies, we observed more pro-inflammation in aging mice both in serum and colonic explants in our study.

Previously, we demonstrated the immuno-modulatory capacity of DDS-1 on lipopolysaccharide-induced human colonic epithelial cells [60]. Similarly, in this study, DDS-1 helped normalize, to some extent, the age-specific pro-inflammatory response and is consistent with previous probiotic studies [61,62]. This could be positively correlated to an increase in abundance of certain beneficial microbes, such as *Akkermansia* and *Lactobacillus* spp., and a decrease in opportunistic microbes, such as *R. gnavus* and *B. acidifaciens*. Increases in the abundance of *Akkermansia* and *Lactobacillus* spp. are reported to be directly correlated with enhanced SCFA production [3,25,27]. Moreover, SCFAs have a multitude of benefits for the host, notably butyrate, which has demonstrated anti-inflammatory effects not only locally but also systemically [47]. Further, SCFAs are known to inhibit NF-κB activation via G protein-coupled receptor 109A receptors and could be responsible for the immuno-modulation observed in this study [52].

To our knowledge, this is one of the first detailed studies comparing intestinal site-specific microbial changes in aging mice with or without probiotic supplementation. Increases in SCFA production, concurrent with immune homeostasis identified in this study, appear to be connected to probiotic-induced microbial and metabolic changes. Consistent with the complex nature of the microbial composition of the mouse, our study confirms differential effects in the caecal and mucosal-associated microbiota. This further suggests the importance of site-specific assessment in the GI microbiota. Our results demonstrate that supplementation with *L. acidophilus* DDS-1 can help modulate shifts in the microbiota in aging mice. In particular, DDS-1 not only enriched beneficial bacteria such as *Akkermansia* spp. and *Lactobacillus* spp. in the intestine but also reduced opportunistic bacteria such as *R. gnavus* and *B. acidifaciens*. Altogether, DDS-1 reduced low-grade inflammation by the release of intestinal microbiota-induced-SCFAs such as butyrate. Further studies, including clinical investigation, are needed to confirm these outcomes. The clinical relevance of this study is that by selectively promoting beneficial bacteria, *L. acidophilus* DDS-1 supplementation could be an important dietary strategy to counteract aging-associated dysbiosis.

## Supplementary Materials

The following are available online at https://www.mdpi.com/2072-6643/11/6/1297/s1, Table S1: List of volatile fatty acids (VFAs) identified in the young control group (YC), young probiotic group (YP), aging control group (AC) and aging probiotic group (AP). The concentrations of VFAs in the caecal content (samples were measured by GC-MS analysis from caecal contents at the end of the study. * *p*  <  0.05 (one-way ANOVA with Tukey’s post-hoc test and data are expressed as the mean ± SEM)., Table S2: List of volatile fatty acids (VFAs) identified in the young control group (YC), young probiotic group (YP), aging control group (AC) and aging probiotic group (AP). The concentrations of VFAs in the mucosal content (samples were measured by GC-MS analysis from mucosal contents at the end of the study. * *p*  <  0.05 (one-way ANOVA with Tukey’s post-hoc test and data are expressed as the mean ± SEM).
