# Supplementary material for: Lactobacillus acidophilus DDS-1 Modulates Intestinal-Specific Microbiota, Short-Chain Fatty Acid and Immunological Profiles in Aging Mice
Source: Nutrients. 2019 Jun 7;11(6):1297. doi: 10.3390/nu11061297 (PMC6627711; doi:10.3390/nu11061297)
Supplement: Supplementary file 1 [file nutrients-11-01297-s001.pdf]

Table 1. List of volatile fatty acids (VFAs) identified in the young control group (YC), young probiotic group (YP), aging control group (AC) and aging probiotic group (AP). The concentrations of VFAs in the cecal content (samples were measured by GC-MS analysis from cecal contents at the end of the study. *$p < 0.05$ (one-way ANOVA with Tukey's post-hoc test and data are expressed as the mean ± SEM).

Volatile fatty acids	AC	AP	YC	YP
Isobutyl acetate	11.47 ± 3.50	6.9 ± 2.33*	7.7 ± 2.32	10.92 ± 0.82*
Isobutyl ether	2.46 ± 0.36	2.43 ± 0.36	2.14 ± 0.41	2.83 ± 0.56
d-Proline, N-methoxycarbonyl-, heptyl ester	1.36 ± 0.26	1.35 ± 0.28	1.17 ± 0.03	1.58 ± 0.85
2-Propen-1-ol, 2-methyl	2.05 ± 0.20	1.93 ± 0.25	1.59 ± 0.22	2.28 ± 0.48
Carbonic acid	8.69 ± 1.69	8.59 ± 0.56	7.02 ± 1.31	8.40 ± 1.43
Ethanedioic acid	0.99 ± 0.22	0.68 ± 0.28	0.77 ± 0.13	0.57 ± 0.11
Butane, 1,1-dibutoxy	0.51 ± 0.08	0.50 ± 0.06	0.64 ± 0.22	0.67 ± 0.16
D-Alanine, N-allyloxycarbonyl-, nonyl ester	1.06 ± 0.17	0.98 ± 0.12	0.78 ± 0.10	1.08 ± 0.18
L-Isoleucine, N-isopropyl-, methyl ester	0.63 ± 0.11	0.59 ± 0.07	0.46 ± 0.07	0.65 ± 0.10
Propanedioic acid, methyl-, dibutyl ester	0.08 ± 0.05	0.10 ± 0.04	0.06 ± 0.03	0.08 ± 0.02
Succinic acid, 3-chlorophenyl 4-methoxybenzyl ester	1.62 ± 0.67	1.07 ± 0.18	0.63 ± 0.29	0.92 ± 0.32
d-Proline, N-isobutoxycarbonyl-, isobutyl ester	0.93 ± 0.57	0.33 ± 0.03	0.36 ± 0.25	0.21 ± 0.02
Carbonic acid, monoamide, N-propyl-N-(hept-2-yl)-, butyl ester	0.88 ± 0.40	0.14 ± 0.03*	0.18 ± 0.07	0.16 ± 0.05
l-Norvaline, N-isobutoxycarbonyl-, isobutyl ester	1.28 ± 0.60	0.30 ± 0.09	0.38 ± 0.07	0.36 ± 0.02
Benzeneacetic acid, 2-methylpropyl ester	0.03 ± 0.02	0.05 ± 0.04	0.04 ± 0.05	0.03 ± 0.01

Table 2. List of volatile fatty acids (VFAs) identified in the young control group (YC), young probiotic group (YP), aging control group (AC) and aging probiotic group (AP). The concentrations of VFAs in the mucosal content (samples were measured by GC-MS analysis from mucosal contents at the end of the study. *$p < 0.05$ (one-way ANOVA with Tukey's post-hoc test and data are expressed as the mean ± SEM).

Volatile fatty acids	AC	AP	YC	YP
Isobutyl acetate	12.58 ± 9.39	16.26 ± 3.42	8.69 ± 11.67	12.67 ± 5.23
Isobutyl ether	5.15 ± 2.86	7.12 ± 2.72*	3.01 ± 0.15	5.1 ± 1.63
d-Proline	2.88 ± 1.56	4.09 ± 1.76*	1.62 ± 1.54	2.90 ± 0.85
2-Propen-1-ol, 2-methyl	4.14 ± 2.31	5.75 ± 2.4	2.17 ± 2.46	4.20 ± 1.36*
Carbonic acid	17.15 ± 12.72	21.43 ± 8.01*	9.01 ± 9.44	17.17 ± 6.88*
Ethanedioic acid	1.22 ± 0.77	1.22 ± 0.36	0.47 ± 0.56	1.42 ± 0.96*
Butane, 1,1-dibutoxy	1.06 ± 0.50	1.59 ± 0.43	1.08 ± 1.22	1.31 ± 0.13
D-Alanine, N-allyloxycarbonyl-, nonyl ester	2.27 ± 1.36	3.31 ± 1.56	1.22 ± 1.49	2.25 ± 0.92
L-Isoleucine, N-isopropyl-, methyl ester	1.36 ± 0.77	2.01 ± 0.93	0.73 ± 0.90	1.36 ± 0.54
Propanedioic acid, methyl-, dibutyl ester	0.50 ± 0.19	0.45 ± 0.30	0.13 ± 0.15	0.62 ± 0.05
Succinic acid, 3-chlorophenyl 4-methoxybenzyl ester	1.72 ± 0.84	1.07 ± 0.18	0.74 ± 0.88	2.22 ± 1.22
d-Proline, N-isobutoxycarbonyl-, isobutyl ester	0.90 ± 0.20	0.83 ± 0.50	0.13 ± 0.10	0.67 ± 0.14
Carbonic acid, monoamide, N-propyl-N-(hept-2-yl)-, butyl ester	0.17 ± 0.05	0.22 ± 0.05	0.06 ± 0.05	0.25 ± 0.23
l-Norvaline, N-isobutoxycarbonyl-, isobutyl ester	0.46 ± 0.40	0.48 ± 0.20	0.17 ± 0.07	0.54 ± 0.40
Benzeneacetic acid, 2-methylpropyl ester	0.02 ± 0.01	0.17 ± 0.05	0.06 ± 0.05	0.25 ± 0.23
